# Trends in Ischemic Mitral Regurgitation Following ST-Elevation Myocardial Infarction Over a 20-Year Period

**DOI:** 10.3389/fcvm.2021.796041

**Published:** 2022-01-13

**Authors:** Leor Perl, Tamir Bental, Katia Orvin, Hana Vaknin-Assa, Gabriel Greenberg, Pablo Codner, Yaron Shapira, Mordehay Vaturi, Alexander Sagie, Ran Kornowski

**Affiliations:** ^1^Department of Cardiology, Rabin Medical Center – Beilinson Hospital, Petach Tikva, Israel; ^2^Affiliated to Sackler Faculty of Medicine, Tel Aviv University, Tel Aviv, Israel

**Keywords:** ischemic mitral regurgitation, myocardial infarction, primary percutaneous coronary, remodeling, ST-segment elevation myocardial infarction

## Abstract

**Background:** Ischemic mitral regurgitation (IMR) is a common complication of acute ST-elevation myocardial infarction (STEMI). Little is known regarding the impact of IMR over a long period of follow up.

**Methods:** Of 3,208 consecutive STEMI patients from a prospective registry, full echocardiographic information was available for 2,985 patients between the years 2000 and 2020. We compared the two decades- 2001 to 2010 and 2011 to 2020, and assessed for the presence of IMR at baseline, 3 (range 2–6) months and 12 (range 10–14) months after the index event.

**Results:** One thousand six hundred and sixty six patients were included in the first decade, 1,319 in the second. Mean patient age was 61.3 ± 12.3 years, 21.1% female patients in the first decade vs. 60.9 ± 12.0 years and 22.2% female in the second (*p* = 0.40 and *p* = 0.212, respectively). Rates of moderate IMR or above during the index admission were 17.2% in the first period and 9.3% in the second one (*p* < 0.001). After 3 months, the rate of IMR was 48.5% for those who suffered from IMR at baseline, vs. 9.5% for those without IMR at baseline (HR- 4.2, *p* < 0.001). Death rates for those with moderate IMR or above were 14.7% and 17.8% after 1 and 2 years, respectively, vs. 7.3 and 9.6% for those without (*p* < 0.001 for both). IMR was associated with 1 year mortality in multivariate analysis (HR-1.37; 1.09–2.20, *p* = 0.009), as well as in propensity score matched analysis (HR 1.29; CI: 1.07–1.91; *p* < 0.001).

**Conclusions:** IMR is a common complication following acute STEMI, impacting prognosis. Rates of IMR have declined significantly over the years.

## Introduction

**Figure 1:**
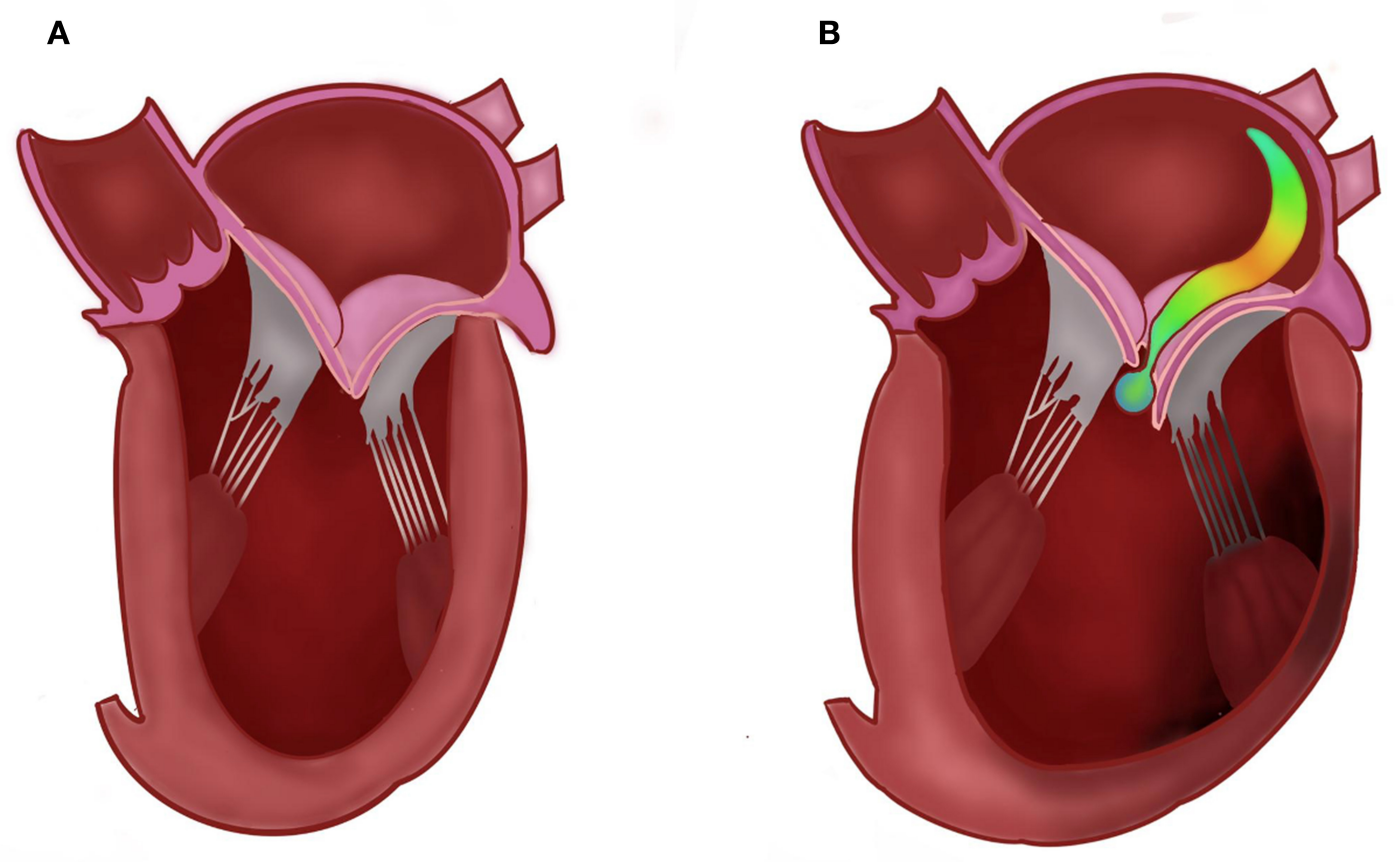
Normal **(A)** vs. ischemic mitral regurgitation **(B)**, demonstrating an eccentric jet due to left ventricular remodeling, displacement of the papillary muscles and tethering of the leaflets.

Ischemic mitral regurgitation (IMR, Figure 1) is a common complication of myocardial infarction (MI) and is caused by left ventricular (LV) remodeling affecting the mitral valve apparatus (1). The estimated incidence of IMR is 11–59% following MI, while moderate to severe IMR appear in 6.3–12.0% of the cases (2–4). The presence of IMR is of grave importance, as it increases the risk for the development of symptomatic heart failure as well as mortality (3, 5–7).

Little is known regarding the natural course of IMR and the tendency of acute IMR to remain permanently. Previous studies suggested that shorter onset-to-reperfusion time and non-total occlusion were found to be independent predictors of early improvement of IMR, whereas higher cardiac biomarker levels, older age, global longitudinal strain and global LV infarct extent were found to have a negative impact in the chronic phase (5, 8). In addition, in some studies IMR was more frequent in patients with an inferior infarction compared with an anterior infarction (9, 10). However, many of the studies have been published based on a limited cohort, both in terms of number of patients and the observation period.

Our aims, therefore, were to investigate the rates of IMR over a 20 year period, based on a large prospective registry of patients suffering from STEMI treated by primary percutaneous coronary intervention (pPCI), in order to study the natural course of IMR following the event.

## Materials and Methods

### Study Design

The present study is based on a prospectively collected STEMI registry from Rabin Medical Center in Petach Tikva—Israel, which includes two campuses—Beilinson and Hasharon hospitals. The registry includes consecutive patients suffering from STEMI who were treated with pPCI from January 2001 through December 2020. The data is continuously entered into an ongoing registry for purposes of recording and monitoring patient-related parameters, clinical events, and angiographic findings.

Of the patients in the registry, we included all patients who have had echocardiographic information during the initial admission, including a proposed mechanism for mitral regurgitation. Patients were excluded if they were treated with thrombolysis instead of pPCI (<1% of cases), if they had known IMR prior to the index event or if they were found to have primary mitral regurgitation prior to MI diagnosis. We then collected information on the echocardiographic exams they have had in the first year, in two more time periods: 3 months (range 2–6 months, period 2) and 12 months (range 10–14 months, period 3). Comparisons were made between the two decades: from January 2001 through December 2010 and from January 2011 through December 2020. The study protocol was approved by the local Institutional Review Board.

### Interventional Procedure

All patients provided explicit written informed consent to undergo cardiac catheterization. Pre-catheterization treatment consisted of aspirin and unfractionated heparin (70 U/kg). Clopidogrel 300 or 600 mg, prasugrel 60 mg, or ticagrelor 180 mg was administered as a loading dose before or immediately after PCI. The utilization of glycoprotein IIb/IIIa inhibitors (GP2b3a) and choice of stent, as well as other therapeutic modalities such as mechanical thrombectomy and distal protection devices, were left to the discretion of the primary operator. All stents were implanted with moderate-to-high deployment pressure (12 to 16 atm). All patients received dual antiplatelet therapy with aspirin 100 mg daily and a thienopyridine (clopidogrel, prasugrel, or ticagrelor) for at least 12 months after PCI.

### Endpoints

Immediate and in-hospital events were prospectively collected in the institutional database. During follow-up, patients completed standardized questionnaires for clinical events either by telephone or in the outpatient clinics at 6 month intervals. When indicated, records from peripheral hospitals were acquired to verify the events in the follow-up period. All events were further confirmed and adjudicated by the institutional clinical events adjudication committee. Survival status at follow-up was assessed by review of municipal civil registries at 30 days and 3 years. Clinical outcomes included all-cause mortality and major adverse cardiac events (MACE), which comprised death, MI, target vessel revascularization (TVR), subsequent coronary artery bypass graft (CABG) and renal failure (defined as glomerular filtration rate below 50 ml/min/1.73 m^2^, according to the Modification of Diet in Renal Disease formula).

### Statistical Analysis

Continuous data are summarized as mean and standard deviation (SD) or median and interquartile range (IQR) and were compared using Student *t-*tests or analyses of variance. Categorical variables are presented as frequency and were compared by chi-square or Fisher's exact tests. The normality of variable distributions was assessed using the Kolmogorov–Smirnov test. Time-to-event curves were constructed using the Kaplan–Meier method and compared using log-rank test. Cox regression analyses were performed to identify independent predictors of the primary end point. Covariates for the Cox model were chosen according to their known association with IMR and outcomes, and included age, sex, diabetes mellitus, hypertension, renal failure, peripheral artery disease (PAD), previous CABG, previous PCI, left ventricular ejection fraction (LVEF, for each 1% increase), the 2011–2020-decade, deployment of drug eluting stents, trans radial access and moderate or above MR at baseline. Effect sizes are presented as odds ratios and 95% confidence intervals. Step-wise variable selection of significant univariate predictors (*P* < 0.1) was used to identify variables for inclusion in the multivariate model. Multivariate logistic regression analyses were performed to determine independent predictors of the primary end point, accounting for known baseline cardiovascular risk differences. Finally, due to several differences in baseline characteristics, we compiled a cohort of propensity score matched patients with a 1:1 ratio between the two decades. The propensity score was derived from a multivariate logistic regression model that included the decade of the admission, considered as the independent (outcome) variable, and all baseline clinical characteristics and procedural characteristics as covariates. The propensity score matched cohort was analyzed for the main combined outcome. Author Leor Perl had full access to all the data in the study and takes responsibility for its integrity and the data analysis. All statistical analyses were performed with IBM SPSS statistics V.27 software. A *P* < 0.05 was considered statistically significant.

## Results

**Figure 2:**
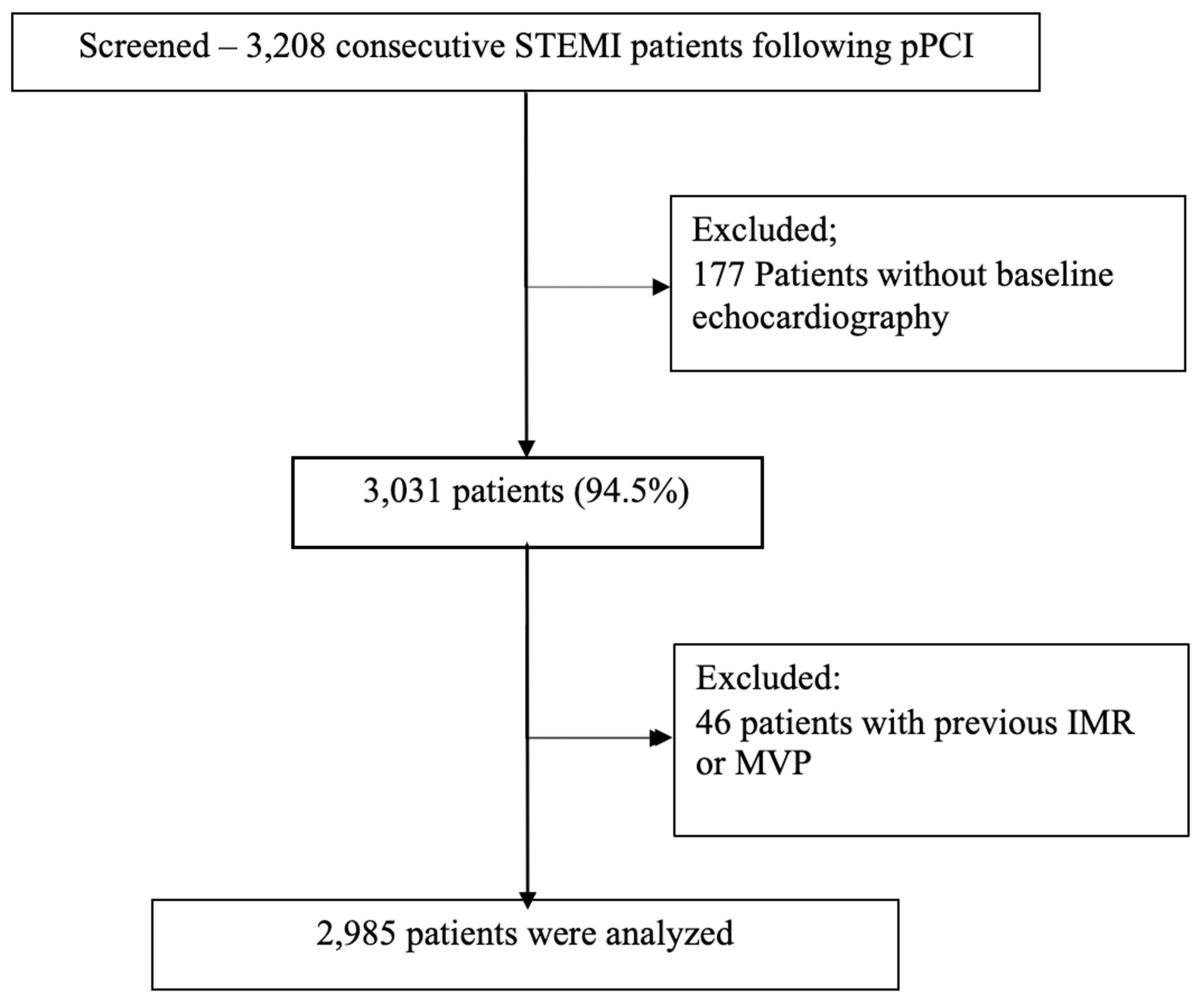
Study cohort.

Of 3,208 patients in the Rabin Medical Center STEMI registry, detailed echocardiographic information existed at baseline for 3,031 (94.5%) of the patients. Following exclusion of patients with known IMR prior to the event, mitral valve prolapse and the other exclusion detailed above, 2,985 patients remained (*n*-1,666 for the first decade and 1,319 for the latter, Figure 2).

Mean patient age was 61.3 years between 2001 and 2010 and 60.9 during the period of 2011–2020 (*p* = 0.40), 22.2% were female patients at the second period vs. 21.1% at the first (*p* = 0.212), 27.6% suffered from diabetes mellitus vs. 27.2% (*p* = 0.634, Table 1). A higher proportion of the patients in the second period also had previous PCI (18.2% vs. 15.0%, *p* = 0.041). Other baseline characteristics were similar (Table 1).

**Table 1:** Baseline characteristics according to decade.

**Parameter**	**Period 1 (2001–2010) *n*-1,666**	**Period 2 (2011–2020) *n*-1,319**	***P*** **-Value**
Age (years)	61.3 ± 12.3	60.9 ± 12.0	0.400
Female Sex (%)	21.1	22.2	0.212
BMI^*^	30.221 ± 9.1	28.884 ± 9.4	0.151
Family History of CAD^†^ (%)	31.9	32.6	0.452
Diabetes Mellitus (%)	27.2	27.6	0.634
Hypertension (%)	49.5	55.3	0.138
Smoking (%)	0.434	0.478	0.392
Hyperlipidemia (%)	0.493	0.517	0.283
Obesity (%)	0.359	0.380	0.356
Renal Failure (%)	0.097	0.080	0.241
Past Stroke (%)	0.056	0.064	0.090
PAD^‡^ (%)	0.051	0.044	0.385
Previous PCI^§^ (%)	0.150	0.182	0.041
Previous CABG|| (%)	0.029	0.035	0.231
Previous MI^#^ (%)	12.3	17.3	0.127
Valvular Surgery (%)	4.5	3.8	0.832

* *BMI, body mass index*;

† *CAD, coronary artery disease*;

‡ *PAD, peripheral arterial disease*;

§ *PCI, percutaneous coronary intervention*;

*^||^CABG, coronary artery bypass graft*;

# *MI, myocardial infarction*.

As for their presentation during the index event, patients in the second decade had lower rates of peak CPK (1569.5 ± 351.5 vs. 2089.7 ± 331.2 mcg/L, *p* = 0.01) and higher LV ejection fraction at baseline (51.3% vs. 48.4%, *p* = 0.031) than patients in the first decade. Patients in the second period were also treated faster (mean presentation to reperfusion time 1.0 h vs. 1.3 h, *p* = 0.041) and by higher rates of transradial PCI (68.2% vs. 21.4%, *p* < 0.001), drug eluting stents (93.2% vs. 38.2%, *p* < 0.001) and prasugrel pharmacotherapy (61.2% vs. 0.0%, *p* < 0.001) than in the first period (Table 2).

**Table 2:** Presentation and procedural details according to decade.

**Parameter**	**Period 1 (2001–2010)** ***n*****-1,666**	**Period 2 (2011–2020)** ***n*****-1,319**	***P*** **-Value**
Systolic blood pressure (mmHg ± SD)	133.4 ± 40.7	135.1 ± 42.5	0.473
Diastolic blood pressure (mmHg ± SD)	77.3 ± 38.2	78.7 ± 37.8	0.383
GFR^*^ (mL/min/1.73 m^2^)	86.3 ± 20.2	86.5 ± 19.4	0.472
Hemoglobin (g/dL ± SD)	13.4 ± 2.4	13.2 ± 3.1	0.274
Platelets (× 10^9^/L. ±SD)	182.1 ± 45.2	186.2 ± 41.6	0.167
Glucose (mg/L±SD)	128.2 ± 47.4	134.2 ± 52.6	0.121
Peak-CPK^†^ (mcg/L±SD)	2089.7 ± 331.2	1569.5 ± 351.5	0.010
Transradial approach (%)	21.4	68.2	<0.001
Symptoms to presentation (hours ± SD)	4.2 ± 2.0	3.9 ± 1.8	0.088
Presentation to PCI^‡^ (hours ± SD)	1.3 ± 0.5	1.0 ± 0.6	0.041
Anterior territory of infarction (%)	48.5%	49.2%	0.425
Inferior territory of infarction (%)	34.2%	33.9%	0.523
Cardiogenic Shock (%)	8.1	4.9	0.091
LVEF^§^ (%)	48.4 ± 12.4	51.3 ± 14.5	0.031
Aspirin (%)	91.0	95.1	0.410
Plavix (%)	88.1	34.1	0.002
Prasugrel (%)	0.0	61.2	<0.001
Ticagrelor (%)	0.0	2.4	0.202
Amines (%)	4.8	2.8	0.448
Number of vessels (N±SD)	1.8	1.8	0.285
Thrombus (%)	69.2	68.4	0.684
Drug eluting Stent (%)	38.2	93.2	<0.001
IABP|| (%)	4.0	3.2	0.420
Temporary Pacemaker (%)	5.3	3.9	0.083
Thrombectomy (%)	13.5	9.8	0.344
Bifurcation (%)	14.8	16.6	0.952
Mean TIMI^#^ pre-procedural (±SD)	1.5 ± 0.9	1.5 ± 0.8	0.960
Mean TIMI post-procedural (±SD)	2.9 ± 0.2	2.9 ± 0.1	0.324

* *GFR, glomerular filtration rate*;

† *CPK, creatine phosphokinase*;

‡ *PCI, percutaneous coronary intervention*;

§ *LVEF, left ventricular ejection fraction*;

*^||^IABP, intra-aortic balloon pump*;

# *TIMI, Thrombolysis in Myocardial Infarction*.

**Figure 3:**
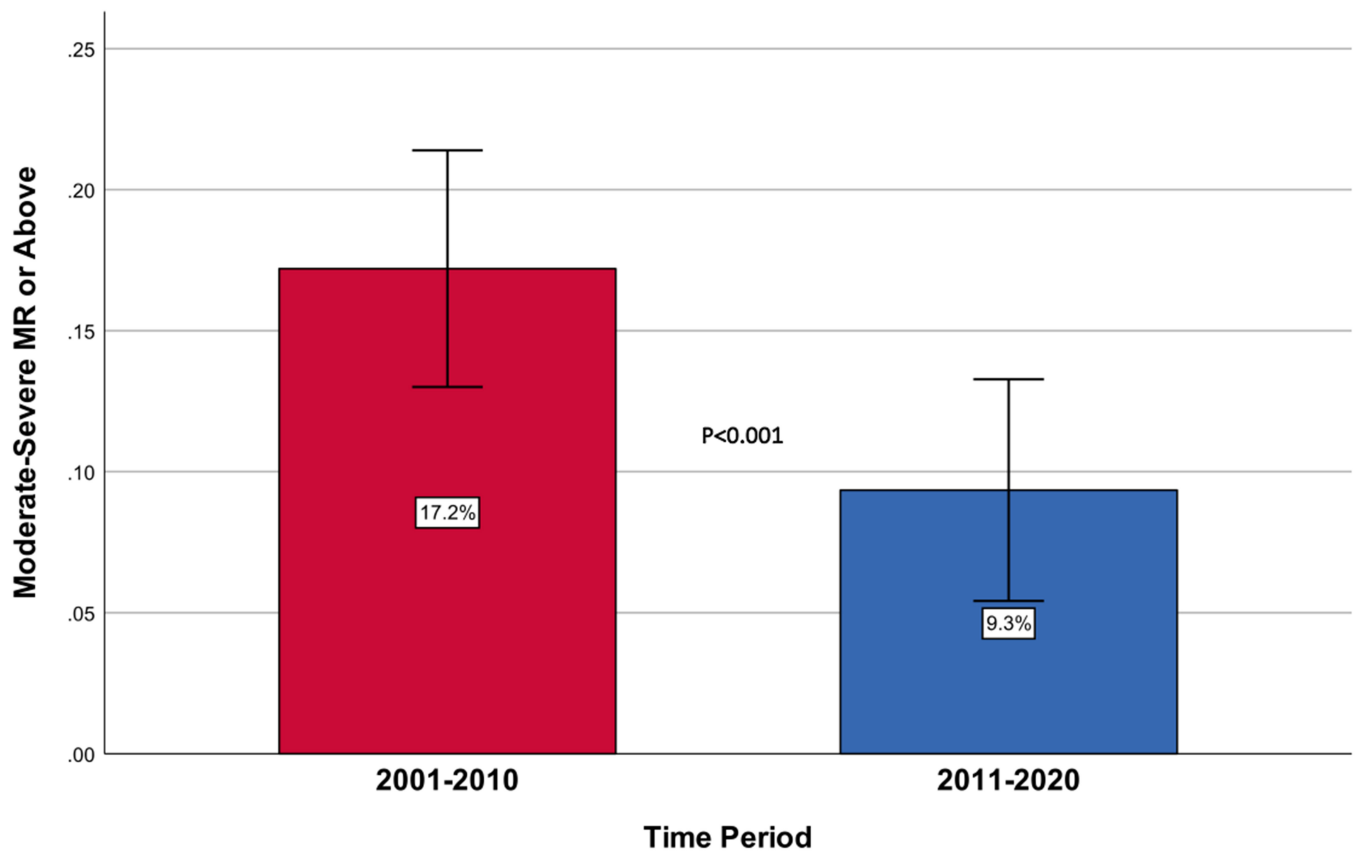
Rates of moderate-severe or above IMR by period.

Rates of moderate IMR or above during the index admission were 17.2% in the first period and 9.3% in the second one (*p* < 0.001, Figure 3). After 3 (range 2–6) months, information was available for 554 of the patients (18.6% of the cohort). The rate of moderate IMR or above was 48.5% for those who suffered from IMR at baseline, vs. 9.5% for those without IMR at baseline (*p* < 0.001). After 12 (range 10–14) months, information existed for 379 patients (12.7% of the baseline). At that point in time, the rate of IMR decreased and was 15.4% for those with significant IMR at baseline, vs. 1.6% for those without (*p* < 0.001).

**Figure 4:**
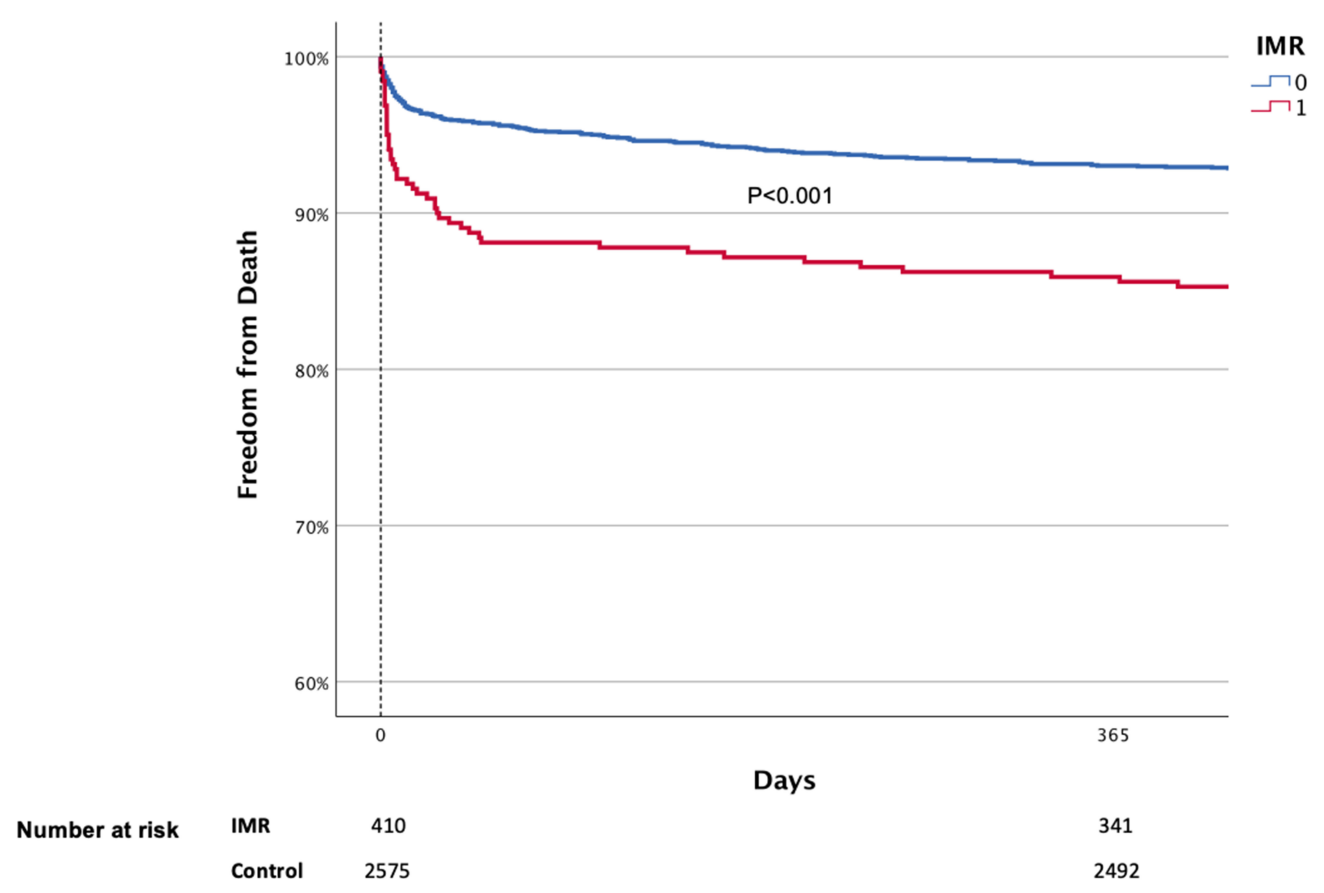
Kaplan Meier curves of survival by IMR.

Death rates for those with moderate IMR or above at baseline were 14.7 and 17.8% after one and two years, respectively, vs. 7.3 and 9.6% for those without (Figure 4, *p* < 0.001 for both). Mortality rates for those who also presented with moderate IMR or above after 3 months was 17.9 and 28.7% after 1 and 2 years, respectively, vs. 10.7 and 17.9% for those without IMR (*p* < 0.001 for both).

**Figure 5:**
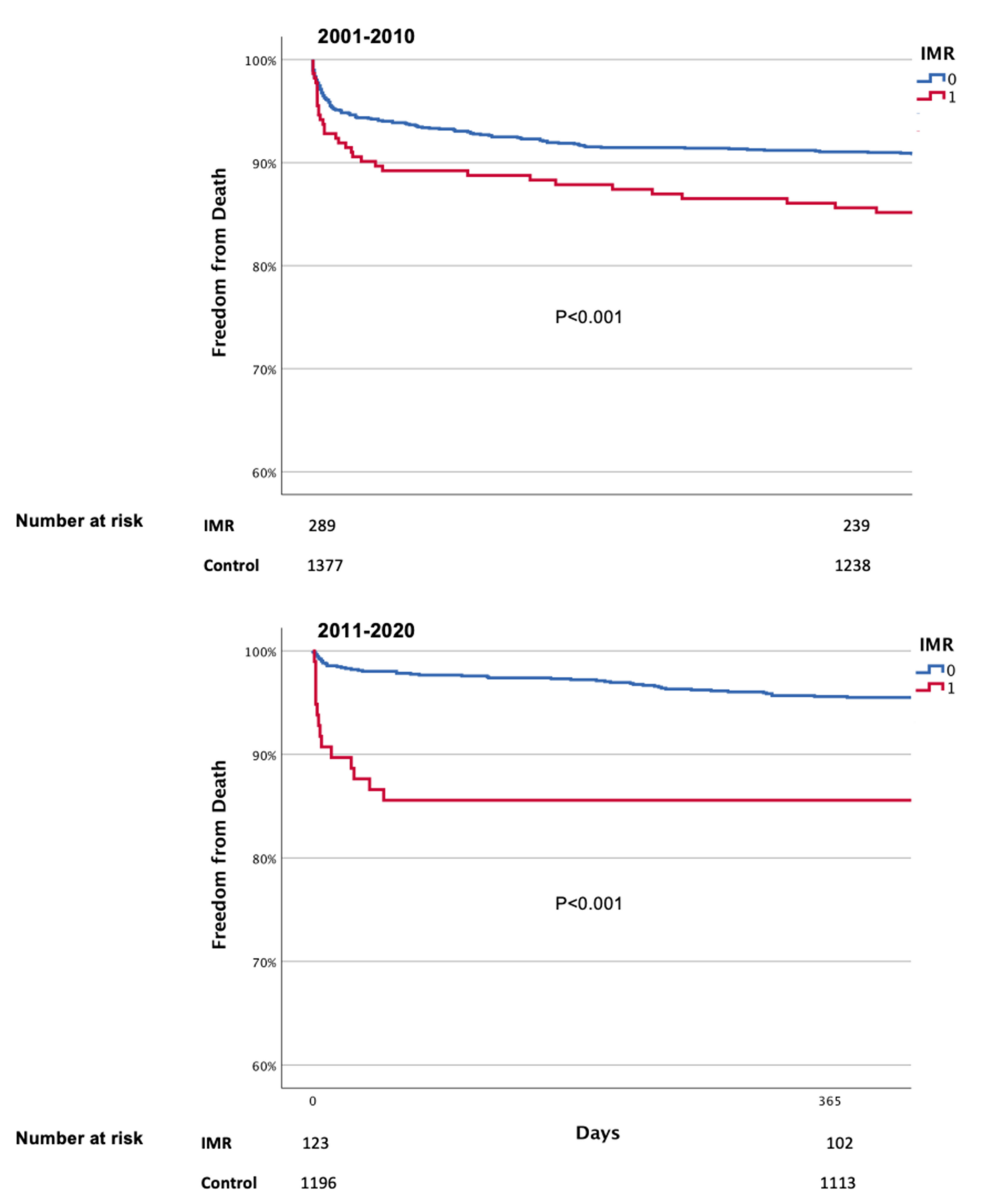
Kaplan Meier curves of survival by decade.

In a multivariate analysis, IMR was found to have an independent impact on 1 year mortality rates (HR-1.37; SD 1.09–2.20, *p* = 0.009), as well as age (HR-1.04; 1.03–1.05, *p* < 0.001), diabetes mellitus (HR-1.58; 1.18–2.12, *p* = 0.002), renal failure (HR-2.79; 2.02–3.85, *p* < 0.001), LVEF (HR-0.920; 0.91–0.93, *p* < 0.001), application of drug eluting stents (HR-0.64; 0.44–0.94, *p* = 0.022), first medical contact (FMC)-to balloon time (HR-1.83; 1.09–2.99, *p* = 0.02) and trans-radial approach (HR-0.83; 0.38–0.99, *p* = 0.044). However, after correcting for these confounding factors, the 2001–2010 decade was not found to negatively impact the risk of 1 year death (HR-1.00; 0.71–1.41, *p* = 0.999, Table 3). Importantly, at both time periods, IMR significantly increased the likelihood of death (Figure 5).

**Table 3:** Multivariate analysis for the risk of 1-year mortality.

**Parameter**	**HR**	***P*** **-value**
Age	1.042 (1.029–1.054)	0.000
Sex (female)	1.217 (0.888–1.668)	0.222
Diabetes Mellitus	1.584 (1.180–2.124)	0.002
Hypertension	0.871 (0.637–1.190)	0.385
Renal Failure	2.788 (2.019–3.849)	0.000
PAD^*^	1.448 (0.951–2.205)	0.085
S/P CABG^†^	1.115 (0.626–1.984)	0.712
Previous PCI^‡^	0.847 (0.580–1.237)	0.390
FMC^§^ to Balloon	1.825 (1.092–2.987)	0.02
LVEF||%	0.920 (0.907–0.933)	0.000
Time period	1.000 (0.711–1.407)	0.999
Drug Eluting Stent	0.640 (0.436–0.938)	0.022
Trans-Radial Access	0.832 (0.384–0.992)	0.044
MR^#^ at Baseline	1.365 (1.094–2.202)	0.009

* *PAD, peripheral arterial disease*;

† *CABG, coronary artery bypass graft*;

‡ *PCI, percutaneous coronary intervention*;

§ *FMC, first medical contact*;

*^||^LVEF, left ventricular ejection fraction*;

# *MR, mitral regurgitation*.

The propensity match score was able to form 288 matched pairs of first- and second-decade patients, showing similar results; Rates of IMR in these two matched cohorts were not significantly different (12.6% in the first decade vs. 9.8% in the second, *p* = 0.08). Following Cox regression, patients presenting with IMR demonstrated higher rates 1 year death than patients without IMR (HR 1.29; CI: 1.07–1.91; *p* < 0.001).

A separate regression was performed to predict the risk of the development of moderate or above IMR at baseline, and following correction for these above factors, the 2001–2010 decade was not associated with risk of IMR (HR-0.93; 0.63–1.22, *p* = 0.08). Factors associated with IMR were LVEF (HR-0.83 for each 1%; 0.46–0.94, *p* < 0.001) and FMC-to balloon time (HR-1.69 for each passing hour; 1.04–2.48, *p* = 0.04, Table 4). The territory of infarct (anterior vs. inferior) did not impact the risk of IMR.

**Table 4:** Multivariate analysis for the risk of IMR.

**Parameter**	**HR**	***P*** **-value**
Age	1.034 (0.914–1.363)	0.071
Sex (female)	1.217 (0.888–1.668)	0.222
Diabetes Mellitus	1.356 (0.872–3.561)	0.002
Hypertension	0.894 (0.527–2.556)	0.855
Renal Failure	2.135 (0.923–5.525)	0.120
PAD^*^	1.241 (0.951–2.213)	0.311
S/P CABG^†^	1.323 (0.515–1.673)	0.329
Previous PCI^‡^	0.924 (0.633–1.592)	0.390
FMC^§^ to Balloon	1.689 (1.038–2.482)	0.040
LVEF||%	0.830 (0.457–0.942)	0.000
Time period	0.930 (0.629–1.219)	0.079
Drug Eluting Stent	0.841 (0.350–1.432)	0.121
Trans-Radial Access	0.910 (0.492–1.549)	0.231

* *PAD, peripheral arterial disease*;

† *CABG, coronary artery bypass graft*;

‡ *PCI, percutaneous coronary intervention*;

§ *FMC, first medical contact*;

*^||^LVEF, left ventricular ejection fraction*.

## Discussion

In this study, we examined the impact of IMR on clinical outcomes in patients presenting with STEMI over a period of two decades, treated at our 2 hospitals in a single combined medical center. We have shown that mortality had changed dramatically over the years, as the time to revascularization, rates of transradial access, P2Y12 and implantation of drug eluting stents have changed. However, the deleterious effect of IMR on death remained profound.

In recent years, there have been significant improvements in the rates of timely revascularization and outcomes for patients suffering from acute coronary syndrome worldwide (11–14). Furthermore, studies assessing the temporal trends following myocardial infarction show a reduction in the rates of mechanical complications over the years. However, when these events occur, they continue to be associated with high mortality rates, which have not improved over the years (15–17).

Mitral regurgitation, whether apparent during the acute or chronic phase of myocardial infarction, increases the rate of adverse events, including all-cause mortality and the risk of the development of congestive heart failure (3, 5–7, 18, 19).

In our study, we have attempted to describe the rates of IMR over a long period of time, and correlate it with mortality. We have seen that rates of IMR have reduced significantly over time, but that in those who present with IMR at baseline, only around half recover after 3 months. We also witnessed a strong impact of IMR on survival, regardless of time period. In our two medical campuses, standard echocardiographic exams are performed immediately after a patient's arrival at the cardiac intensive care unit by skilled sonographers as a protocolized patient care for acute coronary syndrome. In addition, all STEMI patients' clinical course and outcomes are recorded and followed-up for adverse events in a dedicated registry. This allows for the serial follow-up of all patients and comparison of outcomes with echocardiography. Similarly, in the study by Nishino et al. (5), a trend for improvement was witnessed over time in close to 40% of patients, but for those who present with IMR at baseline, the risk for chronic IMR remains. In addition, the presence of IMR in the acute phase worsens prognosis. Also, in similar fashion to the results by Nishino et al. we have found no correlation between the infarction territory and the risk for IMR, as opposed to the outcomes published previously, mostly during the thrombolysis era of STEMI. However, in that study, as well as in the study by Zhang et al. (8), an increased risk of IMR was correlated with older age and higher CPK levels, but in our study these factors did not affect the risk of IMR. Risk factors that were shown to increase the risk of IMR included reduced LVEF and longer FMC-to balloon time. In fact, when correcting for the differences in these factors, the risk of IMR is similar between the years 2001–2010 and 2011–2020.

We have previously shown that in our cohort, the transradial approach improves survival in our PCI patients (20), as was evident in numerous other studies, including in cohorts of STEMI (21–25). Additionally, 2nd generation DES in primary PCI was shown to improve outcomes as compared to 1st generation or bare metal stents (26–28). Both of these factors predicted improved prognosis in our patient population as well, but had no impact on the risk of development of IMR. In our study, the risk for IMR is correlated with a longer FMC-to-balloon time and reduced LVEF. It was previously shown that the extent of global LV infarction extent independently predicts IMR following STEMI (8), mostly due to geometric changes in the mitral valve apparatus with greater displacement of posterior papillary muscle (1). These changes are dependent upon the amount of rescued myocardium by emergency revascularization. The earlier the FMC-to-reperfusion time, the greater the degree of rapid LV improvement is expected. Therefore, improvement in the techniques to achieve timely reperfusion worldwide may continue to reduce the rates of mechanical complications, including IMR, in the years to come. In addition, thanks to advancements in the percutaneous methods to correct functional MR, we may also soon witness a true therapeutic option for IMR directly, in patients with STEMI. New preliminary data now suggests edge-to-edge mitral valve repair may benefit patients with IMR who are hemodynamically unstable (29–31). Conceivably, these and other new therapeutic modalities may potentially become a viable option in reducing the risk for this menacing complication. However, the basic etiology of IMR is ischemia, and it is also possible that more complete revascularization may reduce potential residual ischemia. Whether IMR is a surrogate of myocardial jeopardy or an independent factor for worse prognosis remains to be proven in future trials. Finally, improved adherence to medications attenuating progressive ventricular remodeling, such as beta blockers and angiotensin converting enzyme inhibitors after STEMI may reduce rates of IMR.

### Limitations

In this observational study, we have baseline information, data on the interventional procedure and outcomes for all patients. However, since this data is gathered from a real-world setting, echocardiographic information is missing for a significant portion of the patients after several months. In fact, <20% have full echocardiographic data at the two time points, after 6 and 12 months. We thus are forced to limit the validity of our conclusions regarding the natural course of IMR over a long period of time for each patient, but convincingly adhere to our conclusions related to the findings at baseline, i.e., the impact of IMR at presentation on outcomes over the course of 20 years. In fact, this is the first study to assess the impact the presence and influence of IMR on outcomes over such a long period of time. While examining the impact of changes in practice over a time span of two decades, we have discovered several key factors for improved outcomes and the risk of IMR. These include a shorter FMC to balloon and LVEF. We also employed propensity matching to correct for differences in time periods. These included all factors related to prognosis. However, it is important to mention that Prasugrel was not available at the early time period, but became the most common anti-P2Y_12_ agent in the second period. This was not a factor we could have included in the matching process. Finally, we do not have detailed information on the extent of LV infarction, beyond the extent of cardiac enzymes/biomarkers data. Future studies incorporating data from cardiac magnetic resonance imaging, in particular, may shed more light on the natural course of IMR post STEMI.

## Conclusions

IMR is a common complication following acute STEMI, impacting prognosis, remaining in about half of the patients after 3 months and 15.4% after 12 months among surviving patients. Rates of IMR have declined over the years, mediated by improved rates of early reperfusion, but it remains a significant risk factor for mortality.

## Data Availability Statement

The raw data supporting the conclusions of this article will be made available by the authors, without undue reservation.

## Ethics Statement

The studies involving human participants were reviewed and approved by Rabin Medical Center IRB. Written informed consent for participation was not required for this study in accordance with the national legislation and the institutional requirements.

## Author Contributions

LP and RK: idea conception. LP, TB, HV-A, GG, PC, YS, MV, and AS: data collection. LP and TB: data analysis. LP, YS, and RK: drafting the manuscript. All authors revised the final version and approved it for publication.

## Conflict of Interest

The authors declare that the research was conducted in the absence of any commercial or financial relationships that could be construed as a potential conflict of interest.

## Publisher's Note

All claims expressed in this article are solely those of the authors and do not necessarily represent those of their affiliated organizations, or those of the publisher, the editors and the reviewers. Any product that may be evaluated in this article, or claim that may be made by its manufacturer, is not guaranteed or endorsed by the publisher.

## Abbreviations

IMR: Ischemic mitral regurgitation
STEMI: ST-elevation myocardial infarction
MI: myocardial infarction
LV: left ventricle
pPCI: primary percutaneous coronary intervention
MACE: major adverse cardiac events
TVR: target vessel revascularization
CABG: coronary artery bypass graft
PAD: peripheral artery disease
LVEF: left ventricular ejection fraction
MVP: mitral valve prolapse
CAD: coronary artery disease
GFR: glomerular filtration rate
CPK: creatine phosphokinase
IABP: intra-aortic balloon pump
TIMI: Thrombolysis in Myocardial Infarction
FMC: first medical contact.
